# Biogeographic Patterns in Members of Globally Distributed and Dominant Taxa Found in Port Microbial Communities

**DOI:** 10.1128/mSphere.00481-19

**Published:** 2020-01-29

**Authors:** Ryan B. Ghannam, Laura G. Schaerer, Timothy M. Butler, Stephen M. Techtmann

**Affiliations:** a Department of Biological Sciences, Michigan Technological University, Houghton, Michigan, USA; University of Wisconsin—Madison

**Keywords:** biogeography, microbial ecology, biomarkers, data mining, machine learning

## Abstract

We conducted a global characterization of the microbial communities of shipping ports to serve as a novel system to investigate microbial biogeography. The community structures of port microbes from marine and freshwater habitats house relatively similar phyla, despite spanning large spatial scales. As part of this project, we collected 1,218 surface water samples from 604 locations across eight countries and three continents to catalogue a total of 20 shipping ports distributed across the East and West Coast of the United States, Europe, and Asia to represent the largest study of port-associated microbial communities to date. Here, we demonstrated the utility of machine learning to leverage this robust system to characterize microbial biogeography by identifying trends in biodiversity across broad spatial scales. We found that for geographic locations sharing similar environmental conditions, subpopulations from the dominant phyla of these habitats (*Actinobacteria*, *Bacteroidetes*, *Cyanobacteria*, and *Proteobacteria*) can be used to differentiate 20 geographic locations distributed globally. These results suggest that despite the overwhelming diversity within microbial communities, members of the most abundant and ubiquitous microbial groups in the system can be used to differentiate a geospatial location across global spatial scales. Our study provides insight into how microbes are dispersed spatially and robust methods whereby we can interrogate microbial biogeography.

**IMPORTANCE** Microbes are ubiquitous throughout the world and are highly diverse. Characterizing the extent of variation in the microbial diversity across large geographic spatial scales is a challenge yet can reveal a lot about what biogeography can tell us about microbial populations and their behavior. Machine learning approaches have been used mostly to examine the human microbiome and, to some extent, microbial communities from the environment. Here, we display how supervised machine learning approaches can be useful to understand microbial biodiversity and biogeography using microbes from globally distributed shipping ports. Our findings indicate that the members of globally dominant phyla are important for differentiating locations, which reduces the reliance on rare taxa to probe geography. Further, this study displays how global biogeographic patterning of aquatic microbial communities (and other systems) can be assessed through populations of the highly abundant and ubiquitous taxa that dominant the system.

## Abstract (precis)

Microbes are ubiquitous throughout the world and are highly diverse. Characterizing the extent of variation in the microbial diversity across large geographic spatial scales is a challenge yet can reveal a lot about what biogeography can tell us about microbial populations and their behavior. Machine learning approaches have been used mostly to examine the human microbiome and, to some extent, microbial communities from the environment. Here, we display how supervised machine learning approaches can be useful to understand microbial biodiversity and biogeography using microbes from globally distributed shipping ports. Our findings indicate that the members of globally dominant phyla are important for differentiating locations, which reduces the reliance on rare taxa to probe geography. Further, this study displays how global biogeographic patterning of aquatic microbial communities (and other systems) can be assessed through populations of the highly abundant and ubiquitous taxa that dominant the system.

## INTRODUCTION

There is increasing knowledge of the vast diversity and the abundance of microbes on our planet. However, we are only beginning to understand microbial dispersal and the potential for microbes to exhibit distinct biogeographic patterns. It has been proposed that the selection of microbes in certain locations occurs through various processes such as the environmental conditions (temperature, salinity, pH, etc.), ecological drift, diversification, and dispersal limitation (1–4). Numerous studies have outlined the relative influences of these proposed ecological drivers, which vary drastically across ecosystem type (terrestrial; in soil and sediments, marine and human) (5–9). This has resulted in a lack of consensus as to the seemingly stochastic nature of diversity observed within microbial communities and their geographic distribution.

Previous studies have applied high-throughput sequencing as a means of characterizing the microbial community composition and their underlying global spatial relationships (10–12). It is apparent that under similar environmental conditions, microbial communities can have distinct compositions across space and time (13–15). These efforts, however, have primarily been studied between local or unique habitats (such as extreme environments) (9, 16–19). Presently, the extent of variation within microbial communities on both local and regional spatial scales sharing similar environmental conditions is understudied, despite being an important component to understanding microbial biogeography. Microbial assemblages from aquatic communities surrounding shipping ports are a novel system for microbial ecologists to query biogeography in part because of the similar physiochemical conditions found between both local and regional scales in these ports.

Interfacing this unique, global data set with machine learning (ML) has allowed us to identify stark contrasts in the microbial community composition across a broad geographic range. We were able to observe subpopulations of the highly abundant and ubiquitous microbes of the same phyla that dominate these communities. Portions of the community belonging to the “rare biosphere” have been suggested to constitute much of the diversity across large spatial and temporal scales (18, 20–22) and are often attributed to the underlying distinction of a geographic location. As a result, observing variation in global biogeography through members of dominant taxa might be overlooked, and it may be possible to now explore this through certain machine learning applications. Applying machine learning to questions of biogeography may allow for resolution of fine-scale geographic differences by using a set of data that contains both microbial composition and class labels (geographic location to which the sample belongs) and learns from the relationship between these two to potentially find the microbial taxa which are most associated with a geospatial location (23). Leveraging the abilities of machine learning approaches, distinctions within seemingly similar microbial communities across a global scale may allow for the future prediction or classification of a geospatial location based on a microbial community and could provide insights into the key microbial groups found in distinct geographic locations.

The coupling of cost-effective next-generation sequencing (NGS) technologies with well-established molecular techniques has allowed us to explore machine learning in the context of biology, ecology, and Earth science in unprecedented ways (24–27). Until now, the full potential of using machine learning to understand biogeography has yet to be achieved. This is largely a consequence of limited global microbial data sets with sufficient replication ranging across large spatial and physiochemical gradients that have been processed through standardized methodology. Here, we are seeking to combine high-resolution sequencing with machine learning to observe trends in biodiversity, investigate the potential for there to be biogeographic patterns in the microbial communities of ports, and determine the potential for machine learning to identify patterns in microbial community data not fully appreciated through the use of traditional statistical approaches used in ecology (27, 28).

Here, we investigate the global biogeography of microbial communities found to occupy shipping ports to determine whether there is a biogeographic signal to taxon distribution throughout this system. In determining the underlying distinctions in microbial community structures between these locations, we performed a community analysis of each microbial population from these ports through 16S rRNA amplicon sequencing. Amplicon sequence variants (ASVs) (29) were assigned to provide the highest resolution possible using this marker gene. As a result, we were able to investigate and identify taxon-spatial relationships across large spatial scales, with high resolution, using machine learning. We collected a total of 1,218 marine and freshwater samples from 604 geospatial locations spanning eight countries and three continents to catalogue 20 ports (each with metadata), initiating an expansive ecological study of port-associated microbes. Additionally, this data set provides a foundation for data mining and comparative ecology by accompanying the larger Tara Oceans Project (30) and Global Oceans Sampling Expedition (GOS) (31), with a focus on shipping ports. The aim of this project is to provide the framework to globally observe the process of microbial biogeography.

## RESULTS AND DISCUSSION

### Port sampling and microbial diversity profiling.

**FIG 1:**
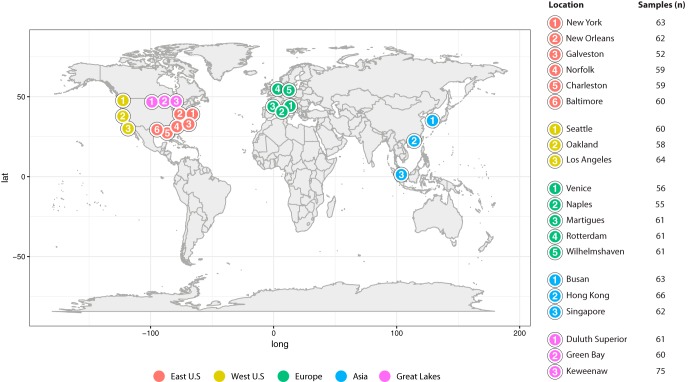
Displayed on the map are the port locations from which samples were collected. All of the sampled ports are binned by the region in which each port is located (East Coast of the United States, West Coast of the United States, Asia, Europe, and the Great Lakes). Sampling depth is displayed by the number of surface water samples collected at each location (*n* = 1,218).

To better understand how microbial community composition is influenced by geospatial location, we used 1,218 surface water samples from 604 locations surrounding ports spanning the Great Lakes, Pacific Ocean, Atlantic Ocean, North Sea, Sea of Japan, South China Sea, Mediterranean Sea, and Adriatic Sea (Fig. 1). These samples were both from marine and freshwater settings and are representative of 20 globally important ports across a range of sizes and ship traffic levels, and they also vary environmentally by pH (5.67 to 9.33), temperature (3.1 to 30.8°C), and salinity (0.040 to 42.35 practical salinity units [psu]).

For these 1,218 samples, 86,411 amplicon sequence variants (ASVs) (29) were identified to assess diversity within microbial communities using the 16S rRNA marker gene. Instead of assigning traditional operational taxonomic units (OTUs), where sequencing reads are clustered by some fixed percent identity threshold, the raw sequence reads were denoised to account for the introduction of any DNA amplification and sequencing errors. By resolving these errors from our next-generation sequencing results, it is possible to dereplicate the reads and examine potentially meaningful information between biological sequences that differ by as little as one nucleotide. This single-nucleotide differentiation in the 16S rRNA marker gene of these bacteria, from all 1,218 samples, allows us to achieve a finer resolution of all the diversity within our data set.

### Characteristics of the dominant microbial taxa of global port microbiomes.

To investigate the distinct biogeographic patterns in the microbial communities of ports, we demonstrated that the taxonomic compositions from our sampling locations vary globally. There were four key bacterial phyla in our data set that dominated throughout all 20 port locations by being both highly prevalent (within 50% or more samples) and highly abundant (those with ≥10% of total 16S rRNA reads with taxonomic assignments at the phylum level). Collectively, these dominant phyla (*Actinobacteria*, *Bacteroidetes*, *Cyanobacteria*, and *Proteobacteria*) accounted for 92% of the total 16S rRNA reads across all samples and contained within them 84% of the total ASVs that were assigned throughout the data set.

**FIG 2:**
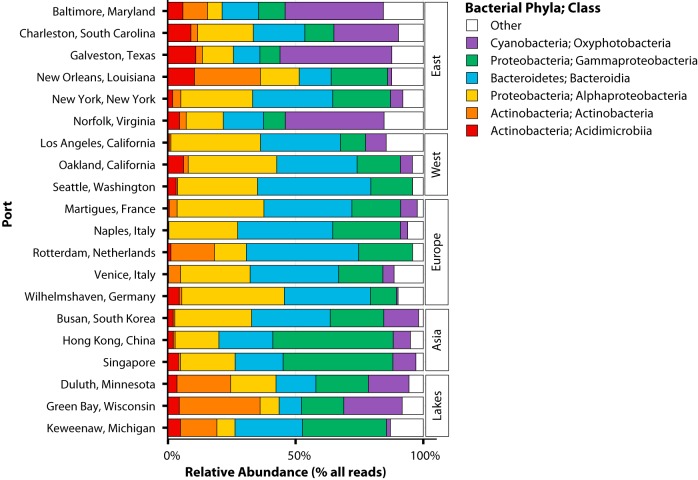
Taxon plot of the composition and relative abundance of the top six dominant bacterial classes across each local port and the region to which they belong based on all 16S rRNA reads. Any bacterial class that did not comprise ≥40% of the ASVs belonging to the top four dominant phyla (*Actinobacteria*, *Bacteroidetes*, *Cyanobacteria*, and *Proteobacteria*) was categorized as “other.”

The following six bacterial classes represented the majority of the variation of these four phyla in their assigned amplicon sequences (e.g., a bacterial class had ≥40% of its respective phylum’s ASV content): *Acidimicrobiia*, *Actinobacteria*, *Bacteroidia*, *Oxyphotobacteria*, *Alphaproteobacteria*, and *Gammaproteobacteria*. *Proteobacteria* was the most abundant phylum overall (42% of total rRNA reads) across all 20 ports and included two of the six most dominant classes (*Alphaproteobacteria* and *Gammaproteobacteria*), which represented 21% and 20% of the total rRNA gene reads, respectively. Together, these six bacterial classes represent 91% of the total 16S rRNA reads and 81% of the total assigned ASVs in this global study and were sufficient to assess the majority of the diversity throughout our sampling locations (see Table S1 in the supplemental material). These six classes were used to demonstrate data set-wide taxonomic composition throughout these globally distributed ports (Fig. 2). Despite such a high prevalence of these classes, there was substantial variation across all locations, with the highest range of variability belonging to the *Cyanobacteria*. For example, the *Oxyphotobacteria* dominated Galveston, TX, in the East Coast of the United States (44% average relative abundance) compared to the two port locations with the lowest abundances for this class, Rotterdam and Wilhelmshaven in Europe (0.01% and 1%, respectively). Additionally, the *Gammaproteobacteria* dominated Hong Kong in Asia (47%) and were least abundant in the East Coast in Galveston, TX, and New Orleans, LA (8%).

10.1128/mSphere.00481-19.8 TABLE S1 Distribution of 16S rRNA reads after diversity profiling (ASV assignment) of dominant taxa. Bacterial classes comprising ≥40% of the ASV content of their respective phyla (assigned ASVs) were chosen as “dominant” classes for downstream analysis. Download Table S1, DOCX file, 0.1 MB. Copyright © 2020 Ghannam et al. 2020 Ghannam et al. This content is distributed under the terms of the Creative Commons Attribution 4.0 International license.

In addition to understanding fine-scale differences between each port, we also sought to determine broader spatial-scale patterns in biogeography of these microbial communities observed from the different regions. We analyzed the variability in the relative abundances of these dominant six classes after grouping each of the 20 port locations into one of the following five geographic regions: East Coast of the United States, West Coast of the United States, Europe, Asia, and the Great Lakes.

**FIG 3:**
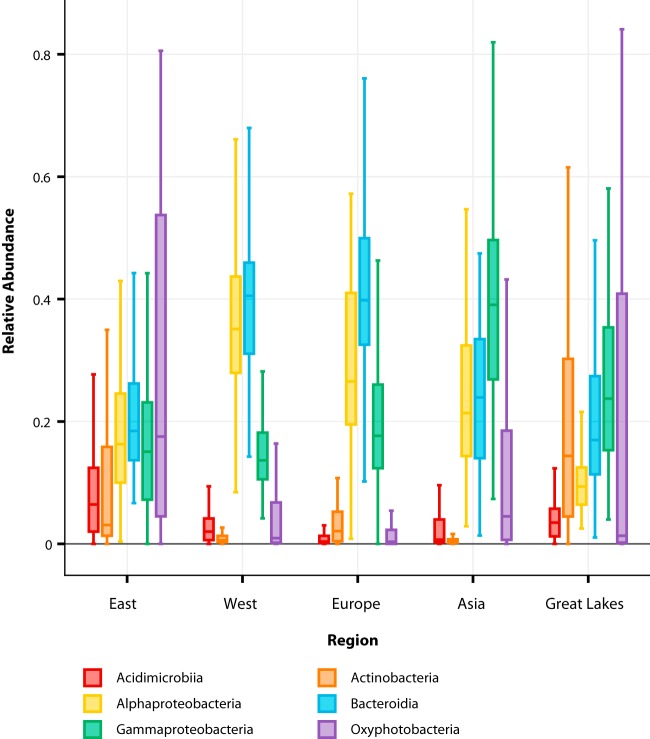
Box plot displaying the differences in community composition of the top 10% most common (dominant) bacterial classes represented as a percentage of relative abundance. Each box represents the interquartile range (IQR) between the first and third quartiles (25th and 75th percentiles, respectively), and the median is represented by the vertical line inside the box. The lines protruding from either side of the box are the lowest and highest values within 1.5 times the IQR from the first and third quartiles, respectively. The relative abundances of all samples of these six dominant bacterial classes in each region are represented by density in Fig. S1. The numbers of samples (*n*) of each region are as follows: East, 355; West, 182; Europe, 294; Asia, 191; and Great Lakes, 196.

Our analysis of these regional taxon-spatial associations shows a substantial abundance of the *Alphaproteobacteria*, *Gammaproteobacteria*, *Bacteroidia*, and *Oxyphotobacteria* compared to the underrepresented *Acidimicrobiia* and *Actinobacteria* across all regions (Fig. 3 and S1). Notably, the Great Lakes have a much higher average relative abundance of *Actinobacteria* (19%) than do the other regions (average relative abundance, <10%). The *Alphaproteobacteria* predominate in the West United States (35%) and have the lowest representation in the Great Lakes (11%). The *Oxyphotobacteria* are more abundant in the samples from the East United States (median relative abundance, 17%) than the lowest median relative abundance belonging to samples from Europe (0.3%). Excluding *Actinobacteria* in the Great Lakes and *Oxyphotobacteria* in Europe and the West Coast of the United States, the six dominant classes had an average relative abundance of >10% across all regions (Fig. 3).

10.1128/mSphere.00481-19.1 FIG S1 Violin plot of relative abundances. Shown are the density and distribution by relative abundances of the six dominant bacterial classes within all samples (*n* = 1,218) and for each region. The wider the distribution, the more samples share similar relative abundances for that taxa. The shape is estimated via a kernel density estimation. Download FIG S1, EPS file, 2.1 MB. Copyright © 2020 Ghannam et al. 2020 Ghannam et al. This content is distributed under the terms of the Creative Commons Attribution 4.0 International license.

### Machine learning uncovers the biogeographic component of microbial communities.

Microbial data are known to be both highly dimensional and compositional (32, 33), and in many cases, the microbial features of the data set are shared between categories to which they belong (e.g., sample type). As a result, many machine learning techniques are often a good approach for understanding how microbial count features of a data set correlate to each other and to a dependent variable (outcome). Compared with the typical statistics used throughout ecology, biogeography, and Earth sciences (33–36), machine learning offers a robust, data-driven estimations of the taxon-spatial associations across globally distributed locations.

We first display the potential to differentiate spatial locations from microbial community data with a multivariate discriminant technique (analysis of similarity [ANOSIM]) applied to both local (all 20 ports) and regional (five regions) scales to assess the ANOSIM in beta diversity. There were more similarities in the microbial communities between the five regions than between the 20 local locations (ANOSIM for regions, |*R|* = 0.609, *P < *0.001; for local port locations, |*R|* = 0.905, *P < *0.001 for Bray Curtis dissimilarity; Fig. S2), where a higher |*R|* value suggests more dissimilarity between communities on the regional or local spatial scale. Similar performance was observed for additional distance metrics (Table S2).

10.1128/mSphere.00481-19.2 FIG S2 ANOSIM plot displaying the dissimilarity between and within local locations (ports) and regions to the microbial communities sampled from them (via Bray-Curtis dissimilarities with 999 permutations). The horizontal line in each box indicates the median; the bottom half of the box indicates the 25th percentile, and the top indicates the 75th percentile. Whiskers project to the most extreme data point, and the width of each box is directly proportional to the sample size at each port or region. A higher |*R*| value represents more dissimilarity between taxonomy and local port or regional scales and is based on the difference of mean ranks between and within groups. Download FIG S2, EPS file, 1.7 MB. Copyright © 2020 Ghannam et al. 2020 Ghannam et al. This content is distributed under the terms of the Creative Commons Attribution 4.0 International license.

10.1128/mSphere.00481-19.9 TABLE S2 Various dissimilarity indices through ANOSIM to complement the Bray-Curtis metric that was reported. All ANOSIM |*R*| statistics displayed with 999 permutations and at 0.001 significance. Download Table S2, DOCX file, 0.1 MB. Copyright © 2020 Ghannam et al. 2020 Ghannam et al. This content is distributed under the terms of the Creative Commons Attribution 4.0 International license.

Additionally, we assessed the community composition through principal-coordinate analysis (PCoA; using Jaccard distances) to observe patterns in the microbial community composition at the regional scale. This form of unsupervised learning is able to simplify the complexity of high-dimensional data sets while retaining trends within bacterial features by transforming it to fewer dimensions. As expected, given how this is an oversimplification of the observed bacterial diversity, only 13.8% of the variation within these communities across each region could be explained by this technique (Fig. S3).

10.1128/mSphere.00481-19.3 FIG S3 Principal-coordinate analysis (PCoA) plot of the global microbial community. This PCoA displays how much of the total sample variance can be explained in the community of all local port samples (*n* = 1,218) using 3,214 ASVs grouped by region (via Jaccard index). Ellipses were calculated assuming a multivariate *t*-distribution with a confidence level of 0.95. Coordinate points clustered closer to each other have more similar microbial communities. The axes indicate coordinate one (Axis.1) and coordinate two (Axis.2), where the percentages in parentheses explain the variation of the whole bacterial community from all of these regions. Download FIG S3, EPS file, 1.3 MB. Copyright © 2020 Ghannam et al. 2020 Ghannam et al. This content is distributed under the terms of the Creative Commons Attribution 4.0 International license.

Last, we assessed these taxon-spatial relationships through supervised machine learning. We were able to find distinctions in the bacterial community for each of the sampling locations locally (all 20 ports) and regionally (five regions) across our global data set. Using random forests (RF; a form of supervised learning) (37), two independent models were used to classify these local and regional geospatial locations (*Y*) from their microbial community alone. At both local (*Y *= 20) and regional (*Y *= 5) levels, all samples (*n *= 1,218, as observations) were able to be accurately binned into the respective geospatial location from which they were collected with high performance. However, these models had slightly more misclassifications while partitioning microbial communities on a local scale (logarithmic loss [log_loss_], 0.101; accuracy, 0.994) than on a regional scale (log_loss_, 0.045; accuracy, 0.995) (Table S3). Given how these models used the same microbial community structure (3,214 high-resolution bacterial predictors [p]; as ASVs), the difference in performances between local and regional models suggests that while able to perform global spatial-scale classifications from microbial communities alone, there were more differences within the microbial communities between regions than there were locally between ports in the same region.

10.1128/mSphere.00481-19.10 TABLE S3 Machine learning classifier performance index of each taxonomic resolution at either the local port or regional scales. These metrics are reported as the macroaveraged results of the ensemble of random forests tuned by the same hyperparameters. Download Table S3, DOCX file, 0.1 MB. Copyright © 2020 Ghannam et al. 2020 Ghannam et al. This content is distributed under the terms of the Creative Commons Attribution 4.0 International license.

Here, classification performance is observed through a reduction in log_loss_ and its relation to increased accuracy. Model accuracy is the overall proportion of correctly classified samples to the local or regional scale to which they belong. Logarithmic loss (log_loss_) measures the quality of predictions and is the probabilistic confidence of how each sample was classified to its local port or region (*Y*) and works by penalizing the incorrect or uncertain predictions. A low log_loss_ is preferred and reflects the distribution of predictions made on a sample toward the true location to which it belongs and how close each sample (observation) was to being misclassified to incorrect geospatial locations.

Interestingly, our ANOSIM results indicated more dissimilarities in the microbial community locally than between five regions, which is in contrast to the RF models which performed better when binning samples into their respective region rather than their individual port. Multidimensional scaling through principal-coordinate analysis of the global port microbial community composition suggested fewer distinctions in microbial community composition than revealed by modeling through RF. Taken together, these results suggest that the ability for a microbial community to be differentiated on the basis of location is possible through a variety of metrics. However, modeling through RF achieved the highest accuracy in differentiating between samples, suggesting that this form of learning can identify differences in the microbial community better than do many of the standard methods for examining microbial community composition.

### The most abundant microbial taxa can be used to discriminate geospatial locations.

The focus of many studies in microbial biogeography has been toward “rare” indicator species as biomarkers for biogeography, as they are assumed to be present in one location and not another (38). Alternatively, highly abundant taxa are easier to detect, as they can differ from the rare biosphere by many orders of magnitude in abundance (18). Therefore, a more generalizable approach for studying biogeographic patterns of microbes may be to leverage the dominant taxa of a system (39).

**FIG 4:**
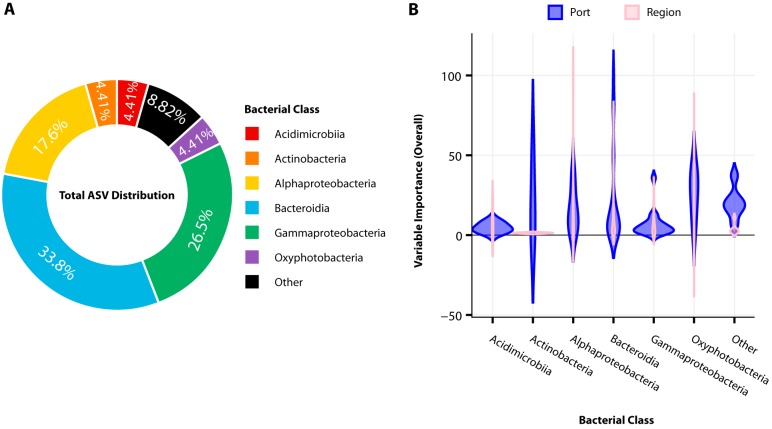
Distributions of shared bacterial classes between machine learning models. (A) Donut chart showing percentages of the 68 shared ASV bacterial predictors after binning the ASVs into the dominant bacterial classes to which they belong. (B) Violin plot (shape via kernel density estimation) of the variable importance by distribution and density of the 68 shared predictor ASVs (overall variable importance, ≥1) and binned by the bacterial class to which the ASV belongs, displayed between both local (port) and regional machine learning models. Here, overall importance for each predictor is the scaled mean decrease in accuracy across all class labels (*Y*) (port, *Y *= 20; region, *Y *= 5). The wider the distribution means, the more similar the importance that ASV predictors belonging to the bacterial class share.

By observing the overall importance of each bacterial ASV predictor used in both models (local and regional), we identified the microbial taxa responsible for the distinction of these globally distributed geospatial locations. There were 342 of 3,214 ASV predictors used in both models that were considered important (overall importance, ≥1 predictor; local, 250 predictors; regional, 92 predictors). Of these predictors, 68 were shared between the two models. These shared bacterial predictors were classified into eight bacterial classes. Notably, 91.17% of these 68 shared predictor ASVs belong to the six most dominant bacterial classes reported previously (Fig. 2), while the remaining two classes (“other”) accounted for only 8.82% (Fig. 4A).

The 68 shared predictors were sorted by their overall importance to show how the dominant bacterial taxa are leveraged to make predictions on both local and regional scales (Fig. 4B). There were more ASVs considered important in the model used to classify a sample into individual ports than to regions, suggesting that more of the overall community was found to be important while identifying distinctions at the highest resolution of spatial scales. The majority of the predictors used in local classifications were distributed across wider ranges of importance, whereas predictors used to make regional classifications are weighted more similarly (Fig. 4B and S4).

10.1128/mSphere.00481-19.4 FIG S4 Frequency polygon of shared bacterial predictors from local and regional models. This displays the 68 shared predictors from our ASV models (from Fig. 4). The ASV predictors were binned into their respective taxonomic classes to show the frequency of features across the variable importance gradient. The *y* axis is the proportion of ASVs belonging to each of the bacterial classes, and the *x* axis is their overall importance to the model. Download FIG S4, EPS file, 0.8 MB. Copyright © 2020 Ghannam et al. 2020 Ghannam et al. This content is distributed under the terms of the Creative Commons Attribution 4.0 International license.

The local model leveraged predictors belonging mostly to the *Bacteroidia* to accurately classify samples, while the regional model used predictors from the *Alphaproteobacteria* (e.g., there is a higher density of predictors in higher overall importance for these classes) (Fig. 4B). *Bacteroidia* and *Alphaproteobacteria* accounted for a large proportion of shared predictors (33.8% and 17.6%, respectively). Between the two models, predictors belonging to the *Oxyphotobacteria* shared similar overall importance and only accounted for 4.41% of the shared predictors. Similarly, the *Acidimicrobiia* also accounted for 4.41% of the shared predictors and had nominal influence as an important predictor, with the highest overall importance of an *Acidimicrobiia* ASV being 8.25 in the local model and 18.56 regional model.

These results align with the distribution of relative abundances of these six dominant classes reported earlier (Fig. 2 and 3). The *Proteobacteria* and *Bacteroidetes* were the two most dominant phyla and accounted for the highest percentage of total sequencing reads, along with the two most dominant bacterial classes belonging to the phyla *Alphaproteobacteria* and *Bacteroidia* (Table S1). *Oxyphotobacteria* had the widest range of variability in relative abundance across all samples. Further, there is a correlation between how these models utilize members of the *Alphaproteobacteria* and *Bacteroidia* and their relative abundances on a local or regional scale. Sequence variants of *Alphaproteobacteria* were considered the most important to the regional model, while variants from *Bacteroidia* were most important to the local model. The choice of members of these classes as being the most important to these models is consistent with the increased differences observed between relative abundances of *Alphaproteobacteria* observed between regions and of *Bacteroidia* observed between local ports (Fig. 2 and 3).

These models used information about members of the most dominant and ubiquitous classes of microbes to make accurate classifications. This suggests that subpopulations in dominant, globally dispersed species are best at explaining geographic patterns in microbial populations. More so, the use of high-resolution ASVs in this study allow for the dissection of fine-scale differences that may represent distinct species, or potentially subspecies, in these populations. These fine-scale differences are able to discriminate between geography at both local and regional spatial scales with high accuracy through machine learning. Observing these regional geographic patterns through abundant taxa has been a challenge largely due to a lack of sufficient sampling density and uniformity in sampling and processing methodology on large spatial scales (9).

### Environmental conditions do not fully explain microbial-spatial diversity on a global scale.

How microbial community composition differs between geospatial locations could be attributed to differences in environmental conditions. It has been suggested that the observed composition of abundant taxa in marine environments is likely a reflection of both historical and current environmental influences (18). A number of environmental variables were measured at the time of sample collection, including conductivity, optical dissolved oxygen (ODO) content, pH, salinity, total dissolved solids (TDS) content, and temperature. The distribution of these six physiochemical variables and their association with each region were analyzed. Each region displayed distinctions between each other for each physiochemical condition other than pH (assessed through analysis of variance [ANOVA], *P < *0.001). (Fig. S5). Further, we correlated the abundance of each bacterial class with these same physiochemical variables for each region (Fig. S6). There are many taxa that are strongly correlated with these measured environmental variables. These findings follow previous work that has shown that the environment plays a key role in selecting for the microbial taxa present in a location in marine environments (40, 41).

10.1128/mSphere.00481-19.5 FIG S5 Comparative ANOVA of physiochemistry across each region. This annotated ANOVA displays the association of each geographic region and six of the measured environmental variables. All comparisons displayed are considered significantly different between each region (*P < *0.001). Download FIG S5, EPS file, 2.3 MB. Copyright © 2020 Ghannam et al. 2020 Ghannam et al. This content is distributed under the terms of the Creative Commons Attribution 4.0 International license.

10.1128/mSphere.00481-19.6 FIG S6 Taxonomic association with region and physiochemistry. Displayed are the correlations between the taxonomic abundances of 38 bacterial classes (after agglomerating all 3,214 ASVs used in our models) and environmental variables at each geographic region. A correlation test (Pearson coefficient |*r*|) was performed, and associated *P* value*s* were adjusted for multiple comparisons for environmental variables (Benjamini-Hochberg). Download FIG S6, EPS file, 1.2 MB. Copyright © 2020 Ghannam et al. 2020 Ghannam et al. This content is distributed under the terms of the Creative Commons Attribution 4.0 International license.

Our classification models were able to accurately discriminate between all 20 ports and five regions by modeling only relative abundances of microbes from the sampled community. To further understand the relationship between environmental conditions and the biogeographic diversity of port microbes, we sought to quantify the amount of variance in the microbial community explained by these measured environmental variables. Across all samples from the 20 port locations, these six physiochemical parameters and their corresponding microbial community composition were used to perform a permutational multivariate analysis of variance (PERMANOVA) (42). This analysis was performed to find the significant conditions that could explain the observed diversity. Conductivity, salinity, and TDS content displayed significant contribution as environmental factors [adonis, Pr(>F) = 0.001, *R*^2^ = 0.833; 0.002 and 0.002, respectively), which cumulatively explains 83% of the variation in microbial diversity within all 20 port locations as one global community. While these environmental variables were considered significant across our data set, the majority of the significance from conductivity could arise from the range of variability in this environmental parameter across samples, for example, since our samples used in analysis come from environments that are either marine water, brackish water (East Coast United States), or freshwater (Great Lakes) (Fig. S5). A constrained analysis of principal coordinates (CAP; Bray-Curtis) was subsequently performed on all six of the physiochemical parameters and microbial community data from the 20 geospatial locations. As expected, given the dimensions of the data set, these six environmental conditions could only explain 22.2% of the observed diversity within this global study (Fig. S7).

10.1128/mSphere.00481-19.7 FIG S7 Constrained analysis of principal coordinates (CAP) of beta diversity and physiochemistry. CAP plot (Bray-Curtis distances) displaying the measured environmental variables and their association to sample variance within the microbial community (grouped by region) of all 3,214 ASVs used in modeling. ANOVA on constrained axis used in this ordination, *F* = 94.66, *P < *0.001. Download FIG S7, EPS file, 1.8 MB. Copyright © 2020 Ghannam et al. 2020 Ghannam et al. This content is distributed under the terms of the Creative Commons Attribution 4.0 International license.

These findings, along with how our ML models perform independent of any physiochemical parameters supplied, show that although the microbial community may be influenced by its environment, the measured environmental information alone is not sufficient to explain the observed biogeographic separation in the microbial community composition.

### Differentially enriched taxa lose discriminant ability across large spatial scales.

To better understand the microbial groups that explain the observed differences in the microbial communities between locations, we employed pairwise differential abundance (DA) analysis. This approach is commonly used in microbial ecology to identify taxa that are overrepresented in a particular sample (43). After assigning all 3,214 ASVs used in this study into 38 bacterial classes, pairwise DA analysis was done comparing each location against all other locations for these 38 bacterial classes in all of the 20 ports in a one-versus-all manner, resulting in 7,220 pairwise comparisons. Our analyses indicated a complement of microbes that are differentially present in these ports around the world.

**FIG 5:**
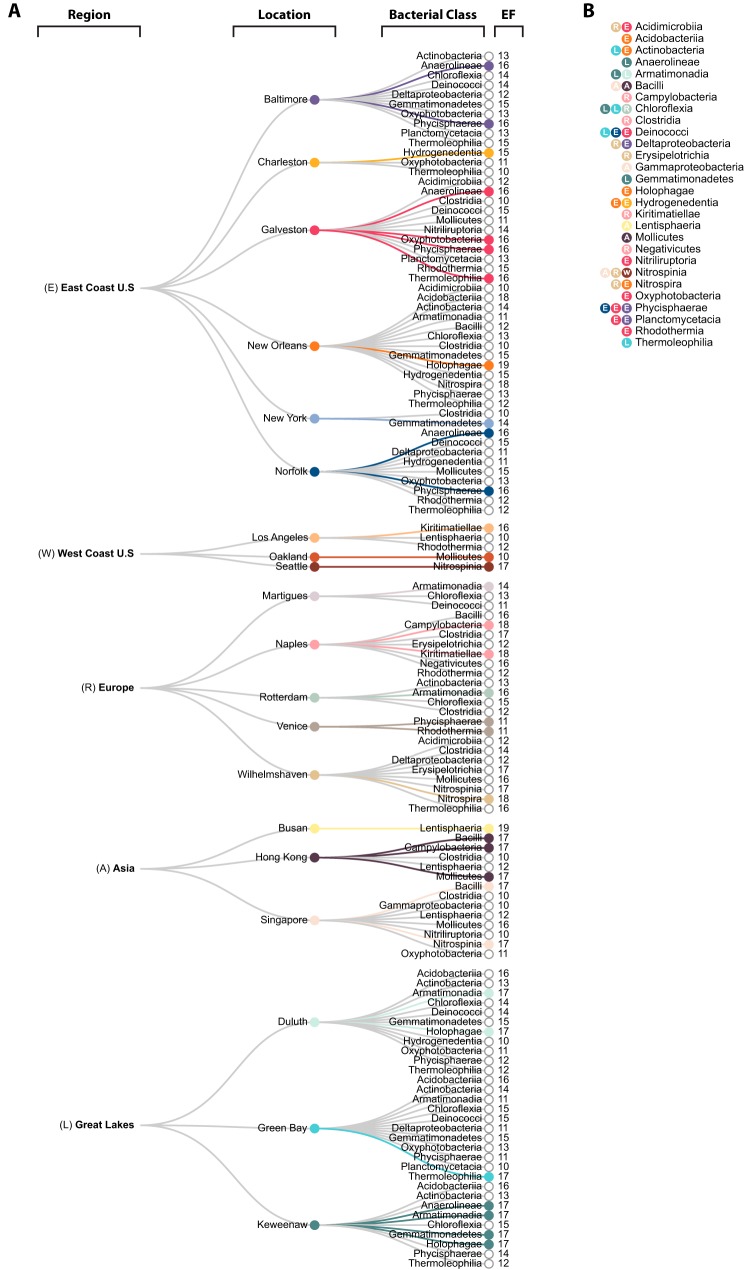
Cluster dendrogram of pairwise comparisons from differential abundance analysis. (A) Dendrogram displaying the 28 unique bacterial classes across all locations with an EF of ≥10. The colored line projecting from each location indicates which class(es) had the highest EF in that location. (B) Displayed for each of the 28 bacterial classes is which location (indicated by color) had the highest total EF for that class along with which region in which it is located (indicated by letter).

A large proportion of these bacterial classes (30/38 [78%]) displayed positive enrichment (log fold change [logFC], ≥ 2; adjusted *P* value [false-discovery rate{FDR}], ≤0.05) in one location over at least one other location. We have termed this the enrichment factor (EF). For example, a bacterial class with an EF of 12 for a location means that the bacterial class has a greater abundance (or is enriched) in that location than in 12 other locations. Our results indicate that each location is composed of a unique consortium of enriched taxa. By assigning an EF, we can ascribe a single bacterial class to a geospatial location that can discriminate it from others. Of the most dominant bacterial classes previously described, only four of the six (*Acidimicrobiia*, *Actinobacteria*, *Gammaproteobacteria*, and *Oxyphotobacteria*) were differentially abundant with an EF of ≥1. *Alphaproteobacteria* and *Bacteroidia* were not considered differentially enriched in any one location more than another (EF, 0), as the relative abundance across each location is too similar to differentiate geospatial location. Of the 30 bacterial classes that were differentially enriched, 28 unique bacterial classes had an EF of ≥10 throughout all 20 locations (Fig. 5A). The distribution of how the 28 unique bacterial classes predominated these locations regionally are as follows: East Coast of the United States, 21 classes; Asia, 9 classes; Great Lakes, 14 classes; Europe, 18 classes; and West Coast of the United States, 5 classes (Fig. 5B). The reported enrichments of the dominant classes at EF of ≥10 were congruent with the relative abundances reported earlier and allow for better differentiation than with abundances alone (Fig. 2 and 3).

Although DA analysis could identify the dominating bacteria in different ports, we observe that for multiple bacterial classes, the same EF was observed at multiple locations (Fig. 5B). Collectively, the use of EF profiles could only differentiate 15 different geospatial locations using 24 bacterial classes, while our machine learning models found 68 subpopulations belonging to eight bacterial classes adequate enough to differentiate all 20 port locations (Fig. 4B and 5B). Machine learning approaches are able to integrate the interaction of multiple features for classification, which is not possible when considering each microbial class independent of each other as DA analysis does. This outlines another strength of the use of machine learning approaches for understanding microbial diversity and biogeography.

The use of enrichment factor and DA analyses did not pick up on some of the most abundant and prevalent taxa that were found to be important for the machine learning classification (*Alphaproteobacteria* and *Bacteroidia*). Instead, low-abundance and low-prevalence taxa were used as discriminators of geospatial location. This observed limitation of DA analysis is consistent with the more generalizable approach of leveraging the highly abundant and ubiquitous taxa for discriminating globally distributed geospatial locations. In the case of ML, as shown with our modeling, accurate classifications are achieved by incorporating the entire community, despite using either all high-abundance taxa, low-abundance taxa, or a mixture of these taxa. This discrepancy between DA analysis and ML may be that the ML models were constructed using ASVs and that the DA analysis was done using an agglomerated table at the taxonomic class level. The use of the class taxon table in the DA analysis was out of the necessity to limit the number of comparisons needed. However, some resolution in the data was lost by agglomerating ASVs into a single class category. Therefore, ML allows for an appreciation of high-resolution microbial count data to observe biogeography.

### The ability to discriminate patterns of biogeography is apparent at the phylum level.

**FIG 6:**
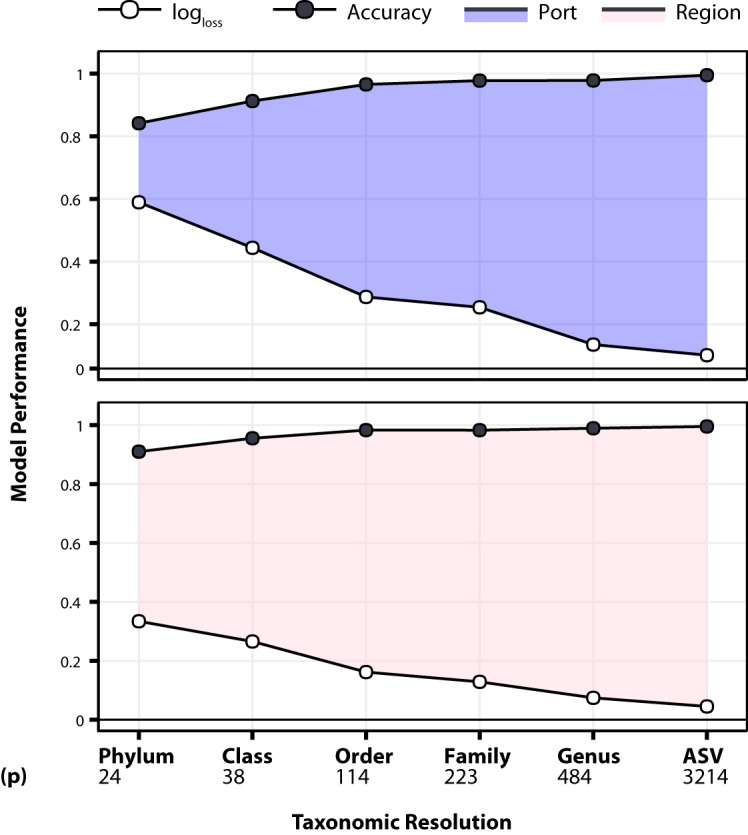
Filled line plot displaying overall logarithmic loss (log_loss_) and accuracy in our machine learning models at each level of taxonomic resolution. Taxonomic resolution is in increasing order on the *x* axis along with the number of predictors (p) used in each model. These models vary in their feature space or number of predictors and class labels (*Y*) (port, *Y *= 20; region, *Y *= 5). All of these multiclass classification models were transformed to 20 one-versus-all or 5 one-versus-all binary classification tasks based on *Y*. The performance metrics log_loss_ and accuracy are expressed as the respective models’ macroaveraged results of the ensemble of random forests tuned by the same hyperparameters.

Our previous machine learning models performed very well at the highest level of resolution (ASVs) (section 3). Therefore, we wanted to determine the ability lower levels of resolution of the microbial community to discriminate geographic location. To decrease resolution, all 3,214 raw sequence variant features (ASVs) from our amplicon reads were binned into their respective taxonomic level (phylum, class, order, family, and genus) and modeled through RF to predict local and regional spatial scales from our samples (*n *= 1,218). Interestingly, the ability for machine learning to establish contrasts in geospatial diversity is apparent at lower taxonomic resolution than expected (Fig. 6).

There were considerable improvements in our performance metrics (log_loss_/accuracy) between spatial scales (local or regional) with models built from the lowest to highest levels of taxonomic resolution (phylum to genus) (Table S3). As taxonomic resolution increased, there was a consistent increase in accuracy and decrease in log_loss_, indicating that our models performed better with increasing taxonomic resolution. Overall, the regional models outperformed the local port models, supporting our earlier findings that learning the biogeography of each sample becomes more challenging as the number of potential geographic locations (*Y*) it could have come from increases (Fig. 6).

Even at the lowest taxonomic resolution of phylum, our models were quite accurate in differentiating geospatial locations locally (log_loss_, 0.58; accuracy, 0.84) and regionally (log_loss_, 0.33; accuracy, 0.90). These accuracies are well above what would be expected for random classifications taking place in our models (based on model kappa, local, 0.83; region, 0.88). The highest reduction of log_loss_ was observed between class-order resolution in both the local and region models (local, 0.16; regional, 0.1) (Fig. 6 and Table S3).

It is notable that of the ASV models which are composed of all ASVs, 3,214 performed better than all lower levels of taxonomy (phylum to genus), where the features arise from agglomerating all 3,214 ASVs into their respective taxonomic levels. This observed trend in increased resolution (e.g., increased predictors [p]) to model performance can be explained by how lower-taxonomic resolutions offer a lower bacterial feature space for which models learn. This finding likely suggests that ML model performance is a result of how much of the microbial community it has available to make data-driven spatial distinctions. Although we observe this resolution-performance scaling, an interesting finding is that at the phylum level, enough differences in the community were observed to bin all samples into their respective port and region with relatively high accuracy. Additionally, we display the ability to agglomerate taxa, which reduces the dimensionality of the data by more than an order of magnitude and results in only a marginal decrease in classification performance (Fig. 6).

**FIG 7:**
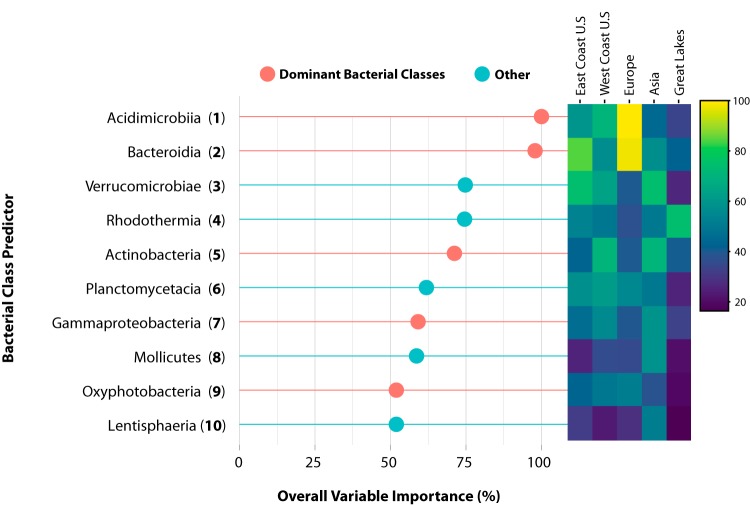
Displayed are the important predictor variables identified by the regional model at taxonomic class-level resolution (Fig. 6) and are taxa that are best at differentiating geospatial location. The red lines indicate that these taxa were among the top six dominant bacterial classes. The overall variable importance is the scaled mean decrease in accuracy for that predictor across all regions (*Y *= 5) and for the ensemble of random forest classifications (these predictors were consistently important across the decision trees in the model). The heat map to the right displays the distribution of overall importance across each region to show the relationship between these bacterial taxa and how they were leveraged by the model to classify samples into each geographic region.

To determine how these models leverage what we know about the underlying structure of the microbial community at these spatial locations, we assessed the regional model at the taxonomic class-level resolution (log_loss_, 0.26; accuracy, 0.95) (Table S3). In this model, the top 10 important bacterial classes and their overall importance across each region were assessed. This reflects how well these bacterial classes could be leveraged by the ML model to help differentiate samples from all 20 ports or five regions. We found that five of the 10 important predictors were among the most dominant classes in this data set, as reported previously, each with an overall variable importance of >50% (Fig. 7). *Acidimicrobiia*, *Bacteroidia*, and *Oxyphotobacteria* were considered most important for samples from Europe (overall importance, 100%, 97.88%, and 51.94%, respectively), while the importance of *Actinobacteria* and *Gammaproteobacteria* was highest for samples from Asia (71.17% and 59.14%, respectively). The overall importance of these taxa in differentiating each region through ML is not directly proportional to the average relative abundance reported for these regions.

It is notable that during these taxon-spatial assessments through ML, Europe has the lowest average relative abundance for *Acidimicrobiia* and the highest for *Bacteroidia* despite the two taxa having the highest variable importance in this region (Fig. 3 and 7). In differentiating regions employing DA analysis through enrichment, we observe the opposite behavior. This could be indicative of these ML models making classifications off a common trend in the microbial abundance (low abundance in one location over others). This finding suggests that caution must be used while inferring associations of a microbial community based on the interpreted importance of taxa in a machine learning model. As such, the variable importance of a taxon is not a direct representation of its biological enrichment in a particular location.

*Alphaproteobacteria* was the only dominant bacterial class that was not considered an important predictor in the bacterial class-level resolution regional model. Interestingly, the absence of this class as part of the top 10 important predictors is consistent with DA analysis results, where *Alphaproteobacteria* could not be considered differentially enriched in any one location more than another. Despite how *Alphaproteobacteria* seemed negligible when observed from both a lower resolution (ML model, class) and DA analysis (Fig. 5 and 7), the ML model utilizing the highest-resolution predictors (ASVs) found *Alphaproteobacteria* to be quite a significant predictor. Sequence variants of *Alphaproteobacteria* were given the highest overall importance in our ASV models regionally (100%), while the same variant was given an overall importance of 42.67% locally (Fig. 4B and S4). The combination of these findings suggests that computationally, these ML models are using different microbial community information at each level of taxonomic resolution to make their predictions and to maintain high accuracy. Biologically, this suggests that biogeographic patterns exist in the presence of distinct ASVs within ubiquitous classes which are present at similar abundances throughout these locations (e.g., ASVs can differentiate location, but the total abundance of the bacterial group to which the ASV belongs is not observably different between locations).

**FIG 8:**
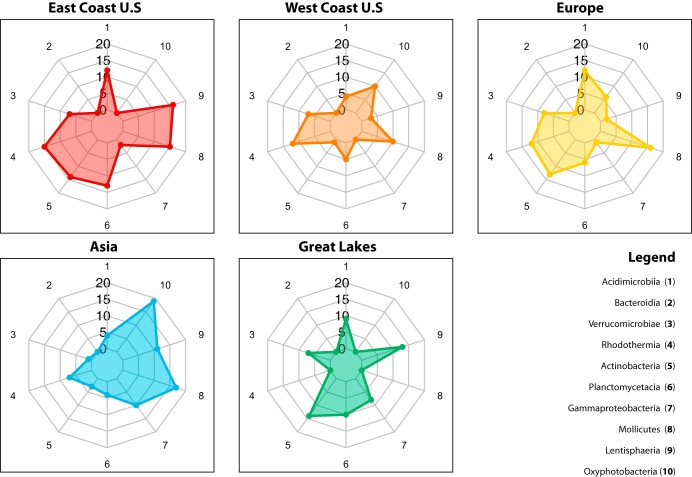
Radar chart of enrichment factors of important predictor taxa. These plots show the enrichment factor (EF) of the top 10 important predictor taxa (Fig. 7) assigned during DA analysis. The vertical axis represents an EF scale of 1 to 20 (as there are 20 local ports). The numbers around the radar charts correspond to the taxa in the legend and indicate those considered most important in their ability to differentiate these geographical regions.

*Gammaproteobacteria* had relatively similar average relative abundance across all regions (Fig. 3). Our machine learning model assigned an overall importance to *Gammaproteobacteria* commensurate to how useful it was to the model for making spatial distinctions across all regions (33.12% to 59.14%) (Fig. 7). This could provide insight into how bacterial taxa with low variability in abundance between locations contribute to machine learning model performance. Similar and notable distinctions between the ML overall importance and DA analysis enrichment metrics were found for the two dominant classes that were not considered differentially enriched (*Bacteroidia* and *Alphaproteobacteria*) yet were assigned an overall variable importance of 100% and 0%, respectively (Fig. 7 and 8).

The ability for us to accurately differentiate between locations using microbial abundance information at high taxonomic levels (albeit low resolution compared to ASVs) suggests that broad differences exist in these microbial communities globally. However, these ML models were slightly more accurate with higher-resolution data, which signifies the importance of geographically distinct subpopulations of the dominant and ubiquitous groups.

This study reports the microbial biogeography of 604 locations belonging to 20 shipping ports distributed globally. We provide a comprehensive data set for the largest study of port-associated microbial communities to date that permits the robust analysis of microbial biogeography across global spatial scales and physiochemical gradients. Accompanying the larger Tara Oceans Project (30) and Global Oceans Sampling Expedition (GOS) (31), this work expands our ability to understand the biogeography of microorganisms in our world’s marine and freshwater aquatic ecosystems.

We identified how much of the complex microbial community structure could be explained in these locations by enrichment through differential abundance analysis and machine learning. Our machine learning models could detect biogeographical patterns in the presence of distinct ASVs within the most ubiquitous and abundant groups (*Actinobacteria*, *Bacteroidetes*, *Cyanobacteria*, and *Proteobacteria*), despite these groups having seemingly relatively equal abundances throughout each location. Distinctions in the microbial community for all 20 ports and five regions into which they group were observable at the lowest level of taxonomic resolution (phylum) and became more granular as we increased to the highest resolution (ASVs) both locally (for phylum, log_loss_, >0.58; accuracy, 0.84; for ASV, log_loss_, 0.10; accuracy, 0.99) and regionally (for phylum, log_loss_, 0.33; accuracy, 0.90; for ASV, log_loss_, 0.04; accuracy, 0.99).

Machine learning could discern how each location contained a distinct composition of sequence variants belonging to these highly abundant taxa better than could commonly used multivariate discriminant techniques and differential abundance analysis. This strongly suggests that between machine learning, commonly used multivariate discriminant techniques, and differential abundance analysis, ML is an optimal approach to uncover biogeographic patterns. Our ML models could appreciate the nature of microbial count data in how both high- and low-abundance bacterial features of the community are distributed across samples and therefore across geospatial locations. As such, these ML models provide a way of finding patterns in diversity and gauging the relative importance of taxa in the overall microbial community at each location on a global scale. Notably, we observed biogeographic patterns in the microbial community composition at a regional scale, where this has previously been a challenge in microbial biogeography across large sampling densities and spatial scales (9).

The work presented here only included samples from a single time point, all during the summer. Therefore, we were unable to address the impact of seasonal changes and/or severe weather events on the observed biogeographic patterns. Since microbial communities are known to vary by season and in response to episodic weather events, we expect there be seasonal impacts on the observed patterns. Analysis of the microbial diversity across two seasons, fall and summer, in the Great Lakes stations used in this study (Duluth, MN; Green Bay, WI; and Keweenaw, MI) shows that the microbial community composition in these locations maintained geospatial taxonomic indicators through these two seasons (44). Future work could include investigation into the temporal dynamics of the observed microbial biogeography of this system. It has been shown previously that community composition shifts in response to seasonal changes can be detected at the level of major taxa (41). We expect that despite the changes in community composition, the dominant and ubiquitous groups would remain throughout seasonal changes. In contrast, taxa that are less abundant and considered rare are seldom retrieved by common molecular techniques that we use on large-scale sampling expeditions (45). Our observation that members of abundant and ubiquitous groups are indicators of geospatial location suggest that these biogeographic patterns may be robust to seasonal changes. Despite longitudinal research showing how dominant bacteria of a system persist throughout the year (40, 41, 45–47), more work is needed to observe exactly how abundant taxa may proportionally stabilize their community composition across large spatial scales and after seasonal changes.

Additionally, severe weather events may perturb the system and may result in transient excursions in microbial community composition. Future studies could investigate the ability of the machine learning classifiers developed in this study to accurately classify samples from a location before, during, and after severe weather events to clarify the persistence of biogeographic patterns despite perturbations. While our study demonstrates the utility of random forests machine learning in modeling and identifying biogeographic patterns, additional work is required to more fully appreciate and model the impact of temporal variation, both seasonal and short term, on biogeographic patterns in microbial communities. Furthermore, while our results suggest that random forests machine learning can be used to more fully appreciate biogeographic patterns, more work could be performed that characterizes the potential for random forests to be applied for modeling of temporal variation in microbial communities.

Although we observed that several existing methods were able to provide insights into our global microbial data set, machine learning appears to provide to deepest insights. This in part may be due to the high-dimensional, highly compositional, and naturally sparse (e.g., contains a lot of zeros) nature of microbial community data (32). There still, however, remains a challenge in ecology to accurately infer associations between microbial communities (48) and, further, their association between geographic locations (39). Despite observing clear trends in biogeography through this robust system, this outlines the urgency to develop statistical methods that are biologically motivated enough to understand the complex taxon-spatial relationships in microbial count data.

## MATERIALS AND METHODS

### Port selection.

Twenty ports were selected to cover globally important ports that varied across a range of environmental conditions, ship traffic, and traffic type (cargo or passenger) and covered multiple continents and various bodies of water. Samples were collected from the following ports: in the Great Lakes at Duluth, Green Bay, and Keweenaw; in the East Coast of the United States at New York (NY), New Orleans (LA), Galveston (TX), Norfolk (VA), Charleston (SC), and Baltimore (MD); in the West Coast of the United States at Seattle (WA) and Oakland and Long Beach (CA); in Europe at Venice and Naples (Italy), Martigues (France), Rotterdam (the Netherlands), and Wilhelmshaven (Germany); and in Asia at Busan (South Korea), Hong Kong, and Singapore.

### Sampling.

The samples used in this study (*n *= 1,218) were collected from 604 locations across eight countries and three continents at a total of 20 ports spanning the Great Lakes, Pacific Ocean, Atlantic Ocean, North Sea, Sea of Japan, South China Sea, Mediterranean Sea, and Adriatic Sea. All samples were collected between May and August 2017. Between 27 and 38 sampling stations were chosen in each port to provide sufficient replication and adequate representation of the range of conditions found within that port. At each station, surface water samples (1 liter) were taken from various locations within that port, each with metadata. Samples were subsequently filtered through a glass fiber prefilter with a 1.6-μm pore size (47-mm diameter) and a 0.2-μm pore-size polyethersulfone (PES) membrane postfilter (47-mm diameter) (Sterlitech Corporation) using a Cole-Parmer Masterflex E/S 115 VAC portable sampler. Filters were placed in 2-ml Eppendorf tubes with 500 μl RNA/DNA shield (ZymoBIOMICS) and stored at ambient temperatures until transported back to the laboratory to be stored at –80°C. Multiparameter data of water quality (conductivity, ODO, pH, salinity, TDS content, temperature, and dissolved oxygen content) along with global positioning system (GPS) coordinates of each sampling site were recorded *in situ* with a YSI ProDSS digital sampling system that was calibrated before each sampling trip.

### DNA extractions.

DNA was extracted from each filter using the ZymoBIOMICS DNA microprep D4305 kit (Zymo Research, Irvine, CA, USA), and for each sample, both the prefilter (1.6-μm pore size, 47-mm diameter) and postfilter (0.2-μm, 47 mm diameter) were cut in half, where one half was to be used in the DNA extraction and the other half stored as a contingency.

### DNA sequencing.

First-stage amplification PCRs were carried out in 25-μl mixtures consisting of 12.5 μl Phusion high-fidelity PCR master mix (Thermo Fisher Scientific, Waltham, MA, USA) containing deoxynucleoside triphosphates (dNTPs) at a concentration of 200 mM each, optimized reaction buffer, 1.5 mM MgCl_2_, and 1 U high-fidelity polymerase per reaction in 96-well VWR polypropylene plates. The primer pair 515f and 926r was used at a concentration of 0.4 μM to amplify a construct that spans the variable regions 4 and 5 (V4–V5) of the 16S rRNA gene (49). The PCR thermal cycler settings were as follows: 95°C for 3 min; 25 cycles of 95°C for 30 s, 55°C for 30 s, and 72°C for 30 s; and 72°C for 5 min. PCR cleanup was performed after first-stage amplification PCR to remove residual primers and excess reagents from PCR mixtures. For this cleanup, we followed the MiSeq library preparation guide (Illumina, San Diego, CA) and deviated from the standard protocol by using AxyPrep Mag PCR cleanup beads (Corning, Big Flags, NY, USA), using 10 mM Tris at a pH of 8 (down from 8.5) and by using 28 μl AxyPrep beads in the second-stage cleanup since the PCR volume was 25 μl (down from 50 μl). Second-stage indexing PCRs took place under the same mixture conditions as first-stage amplification PCR and with primers that contained a unique index sequence for each sample and the Illumina sequencing adaptors. An additional PCR cleanup was done after second-stage PCR, eluting to a final volume of 50 μl. Library preparation and sample pooling were performed according to the MiSeq 16S sequencing library preparation guide (Illumina). The products from the second-stage indexing PCR and subsequent cleanup stages were pooled into a library for sequencing at an equimolar concentration of 10 nM after ensuring that primer contamination was absent or at a minimum using a 2100 Bioanalyzer (Agilent, Santa Clara, CA). Denaturation and dilution of the pooled 16S rRNA gene library were performed according to the MiSeq 600-cycle V3 reagent kit guide (Illumina) to produce a 2 × 300-bp paired-end run. These samples were sequenced over three separate sequencing runs containing 672, 480, and 396 samples, respectively.

### Computational analysis and visualization.

All statistical analysis, machine learning models, and visualization were conducted on a local server (Red Hat Enterprise Linux server 7.3 [Maipo]; 256 Gb of random-access memory [RAM]) and on R environment version 3.5.0 (50) using the following packages and associated dependencies: DADA2 (51), phyloseq (52), DESeq2 (53), hpgltools (54), microbiome (55), microbiomeSeq, vegan (56), caret (57), caretEnsemble (58), and randomForest (59), the visualization packages ggplot2 (60) and plotly, and through rawgraphs.io.

### ASV identification and taxonomic profiling.

Raw 16S rRNA sequencing reads were demultiplexed using the Illumina MiSeq platform. Through the divisive amplicon denoising algorithm (DADA2 package) (51) in R, primer nucleotides were removed, and overlapping paired-end reads were merged, quality filtered, and cleansed of internal standard phiX; to distinguish amplification and sequencing errors from true biological variation in our collected samples, amplicon sequence variants (ASVs) were inferred. To account for learning the inherently different error rates in each of the three separate sequencing runs, samples (672, 480, and 396) from each run were inferred independently (from >100 million bases) so as not to bias the true sequence diversity contained in the final data set of the combined samples. The three independent ASV count tables were merged and then used to resolve and remove chimeric artifacts with higher accuracy as a result of the resolution of ASVs. Traditionally with OTU picking, chimeric sequences are removed in a conservative manner, as closely related sequences are later merged into the same OTU. While using ASVs, a more sensitive removal is accomplished by performing a Needleman-Wunsch global alignment of each sequence, finding bimeras (two-parent chimeras) and localizing combinations from a left and right parent chimera that overlaps the child sequence exactly. From 52,316,084 paired-end input reads, a total of 23,235,684 nonchimeric reads passed our filtering parameters and were used in ASV identification and analysis in this study. We obtained a count table analogous to the generally used OTU table; similarly, our features in this table are composed of the uniquely inferred ASVs that map how many of these amplicon variants were observed in each sample. Taxonomy of ASVs was assigned through DADA2 (51) with a reimplementation of a rapid assignment naive Bayesian classifier that compares our biological sequence variants to a training set of previously accurately classified sequences using the SILVA v132 training set (61, 62).

### Dimensionality reduction and normalization of data.

A series of filtering criteria were applied to the final sequencing count table of 1,514 samples and 117,397 ASVs. Initially, only samples only from open water and those that had >1,000 16S rRNA reads were chosen to be in our data set for microbial community analysis. Additionally, every ASV that was not under the kingdom *Bacteria* was removed, along with a prevalence filtering step to only keep ASVs that were within ≥15 samples (e.g., an amplicon sequence variant had to be present in 15 or more samples from 1,218 total samples). Subsequently, singleton ASVs that either had a quantity of one in any sample or were only present in one sample along with ASVs that summed to zero across all samples were removed, resulting in a data set of 1,218 open-water samples and 3,214 ASV features. The absolute ASV read counts were logarithmized with the standard log_10_(*x* + 1) using the transform function in the microbiome package in R (55); this count table was used for all downstream statistical analysis and machine learning. To simplify downstream visualization, supply count tables with reduced feature dimensions, and compare lower-taxonomic-level model performance against high-resolution ASVs both locally and regionally, phyloseq (52) was used to agglomerate all 3,214 ASVs into their respective levels of taxonomy (phylum to genus).

### Annotation of environmental conditions.

All 3,214 ASVs were used to identify which environmental conditions were considered significant in explaining beta diversity in our microbial community across spatial scales. PERMANOVA (42) was conducted using distance matrices (Bray-Curtis) with 999 permutations in vegan (56), and significance (*P < *0.001) was assessed through F testing based on the sequential sums of squares between the physiochemical parameters chosen and the five geographic regions to which the local ports were assigned. To account for the trends in environmental conditions and their correlation to each region, these same physiochemical parameters were used to annotate an ANOVA of each condition across all regions (*P < *0.001). In order to detect the biotic relationships of the taxa and their association to the six physiochemical parameters, we used our ASVs to identify correlations using Pearson coefficient |*r|* (63), and associated *P* value*s* were adjusted for multiple comparisons for environmental variables (Benjamini-Hochberg). Finally, to define how well these six physiochemical parameters could explain the total sample variance in the microbial community, a constrained analysis of principal coordinates (CAP; Bray-Curtis) was applied to all 3,214 ASVs using vegan (56).

### Analysis of similarity and ordinations.

To show whether the microbial community structures of the 3,214 ASVs were significantly different between local ports and regional ports, ANOSIM (|*R|*) was performed on absolute ASV counts using a Bray-Curtis dissimilarity matrix with 999 permutations. To visualize differences within this community, a principal-coordinate analysis (PCoA) was generated using phyloseq (52) using the ordination function (Jaccard index) and visualized through the plot_ordination function, where ellipses were calculated assuming a multivariate *t*-distribution with a confidence level of 0.95.

### Differential abundance analysis and identification of enrichment factors.

We used the count table that was agglomerated to the class level as a sufficient level of taxonomic resolution to detect differentially abundant taxa between all ports. These data were used to create an experimental design model with hpgltools (54) so that a pairwise contrast could be made for each of 20 locations against the other and across all features (38 bacterial classes), with *n* biological replicates supplied as *n* samples per location, ranging from 52 to 75, with a total of 1,218 samples (Fig. 1; samples [*n*]). These counts were normalized assuming a negative binomial distribution, and a parametric gamma-family generalized linear model fitting scheme was applied over taxon-wise dispersion estimates using DESeq2 (53). Of these 7,220 pairwise comparisons, taxa were only considered differentially enriched and were assigned an enrichment factor (EF) if they satisfied the following conditions: had a logFC of ≥2, had an adjusted *P* value (FDR) of ≤0.05, and were in one location over at least one other location.

### Machine learning.

Our normalized ASV and agglomerated genus, family, order, class, and phylum count matrices were used as input data from which to learn. The same hyperparameters were chosen to ensemble the random forests in caret (57) and caretEnsemble (58) as follows: repeated *k*-fold cross-validation (*k = *10 with 3 repeats) so as to estimate the generalization performance of the models, ntree = 501 (number of trees grown), and a random search for best mtry (the number of predictors sampled at each node); last, input data were centered by removing the mean value of each feature and scaled by dividing nonconstant features by their standard deviation. All models were trained with a multiclass summary function so that macroaveraged results of the ensemble of all random forests tuned by these same hyperparameters could be reported. As these are multiclass classifications, depending on the model type (local, *Y *= 20; regional, *Y *= 5), each model was transformed to either 20 one-versus-all or 5 one-versus-all binary classification tasks. Each model in the ensemble was fit with the same resampling indexes across each *k*-fold.

### Code availability.

All code used for statistical analysis, machine learning, and the figures is available through GitHub (https://github.com/rghannam/portmicrobes).

### Data availability.

The National Center for Biotechnology Information (NCBI) Sequence Read Archive has archived the raw sequencing data and associated metadata used in this study under the accession numbers PRJNA542890 and PRJNA542685. All other relevant data necessary for this workflow or that support the findings of this study are available in the supplemental material, from the corresponding authors upon request, and through GitHub (https://github.com/rghannam/portmicrobes).
