# Supplementary material for: Biogeographic Patterns in Members of Globally Distributed and Dominant Taxa Found in Port Microbial Communities
Source: mSphere. 2020 Jan 29;5(1):e00481-19. doi: 10.1128/mSphere.00481-19 (PMC6992368; doi:10.1128/mSphere.00481-19)
Supplement: TABLE S1 [file mSphere.00481-19-st001.docx]

| Total 16S rRNA reads for dominant phyla | | | 13,733,236 |
| --- | --- | --- | --- |
| Total ASVs assigned during diversity profiling | | | 3,214 |
| **Phylum** | **% Total Reads** | **% Total ASVs** | **Assigned ASVs** |
| Actinobacteria | 12.09 | 7.56 | 243 |
| Bacteroidetes | 26.07 | 29.37 | 944 |
| Cyanobacteria | 11.74 | 4.6 | 148 |
| Proteobacteria | 42.76 | 43.06 | 1384 |
| **Total** | **92.66%** | **84.59%** | **2,719** |
| **Class** |  |  |  |
| Acidimicrobiia | 3.80 | 3.14 | 101 |
| Actinobacteria | 8.15 | 4.01 | 129 |
| Bacteroidia | 25.48 | 28.84 | 927 |
| Oxyphotobacteria | 11.74 | 4.60 | 148 |
| Alphaproteobacteria | 21.56 | 19.60 | 630 |
| Gammaproteobacteria | 20.91 | 20.87 | 671 |
| **Total** | **91.64%** | **81.06%** | **2,606** |
