# Supplementary material for: Biogeographic Patterns in Members of Globally Distributed and Dominant Taxa Found in Port Microbial Communities
Source: mSphere. 2020 Jan 29;5(1):e00481-19. doi: 10.1128/mSphere.00481-19 (PMC6992368; doi:10.1128/mSphere.00481-19)
Supplement: TABLE S2 [file mSphere.00481-19-st002.docx]

| ANOSIM dissimilarity metrics | | |
| --- | --- | --- |
| **Dissimilarity metric** | Local | Region |
|  | \|R\| | |
| Canberra | 0.9607 | 0.6402 |
| Manhattan | 0.5653 | 0.2541 |
| Jaccard | 0.9048 | 0.6092 |
| Jensen-Shannon | 0.9325 | 0.6242 |
