# Supplementary material for: Biogeographic Patterns in Members of Globally Distributed and Dominant Taxa Found in Port Microbial Communities
Source: mSphere. 2020 Jan 29;5(1):e00481-19. doi: 10.1128/mSphere.00481-19 (PMC6992368; doi:10.1128/mSphere.00481-19)
Supplement: TABLE S3 [file mSphere.00481-19-st003.docx]

| **Model resolution** | **Metric** | | | **Predictors** |
| --- | --- | --- | --- | --- |
|  | **Accuracy** | **LogLoss** | **Precision/Recall** | **p** |
| **Local: Y = 20** | | | |  |
| Phylum | 0.84150114 | 0.58928161 | 0.85460672/0.83943254 | 24 |
| Class | 0.91242087 | 0.44398276 | 0.92566468/0.91223214 | 38 |
| Order | 0.9654911 | 0.28712688 | 0.97033929/0.96514087 | 114 |
| Family | 0.97759414 | 0.25411364 | 0.98079497/0.97707738 | 223 |
| Genus | 0.97806738 | 0.1352262 | 0.98106349/0.97784722 | 484 |
| ASV | 0.99478113 | 0.10128259 | 0.99547619/0.995 | 3,214 |
| **Region: Y = 5** | | | |  |
| Phylum | 0.90991625 | 0.33421786 | 0.91091799/0.9037573 | 24 |
| Class | 0.95531278 | 0.2656141 | 0.95568987/0.95368017 | 38 |
| Order | 0.98300733 | 0.16181949 | 0.98246165/0.98374711 | 114 |
| Family | 0.98277012 | 0.1286815 | 0.98300881/0.98234723 | 223 |
| Genus | 0.98930743 | 0.07437649 | 0.98949777/0.99056985 | 484 |
| ASV | 0.99535038 | 0.04514882 | 0.99497995/0.99539333 | 3,214 |
